# Structural characterization of newly-developed Al_79_Ni_5_Fe_5_Y_11_ and Al_79_Ni_11_Fe_5_Y_5_ alloys with amorphous matrixes

**DOI:** 10.1038/s41598-023-48282-3

**Published:** 2023-12-03

**Authors:** Katarzyna Młynarek-Żak, Indu Dhiman, Krzysztof Matus, Mariola Kądziołka-Gaweł, Wojciech Łoński, Adrian Radoń, Tomasz Czeppe, Rafał Babilas

**Affiliations:** 1 Department of Engineering Processes Automation and Integrated Manufacturing Systems, Silesian University of Technology, Konarskiego 18a St., 44-100 Gliwice, Poland; 2 Budapest Neutron Centre, Konkoly-Thege Miklos 29-33 St., Budapest, 1121 Hungary; 3 Materials Research Laboratory, Faculty of Mechanical Engineering, Silesian University of Technology, Konarskiego 18a St, 44-100 Gliwice, Poland; 4 Institute of Physics, University of Silesia, 75 Pułku Piechoty 1 St., 41-500 Chorzów, Poland; 5 Department of Engineering Materials and Biomaterials, Silesian University of Technology, Konarskiego 18a St., 44-100 Gliwice, Poland; 6 Łukasiewicz Research Network, Institute of Non-Ferrous Metals, Sowinskiego 5 St., 44-100 Gliwice, Poland; 7 The Aleksander Krupkowski Institute of Metallurgy and Materials Science, Polish Academy of Sciences, Reymonta 25 St., 30-059 Kraków, Poland

**Keywords:** Metals and alloys, Materials science, Microscopy

## Abstract

The low glass-forming ability of aluminium-based metallic glasses significantly limits their development and preparation. This paper updates the current state of knowledge by presenting the results of structural studies of two newly-developed Al_79_Ni_5_Fe_5_Y_11_ and Al_79_Ni_11_Fe_5_Y_5_ alloys with a reduced aluminium content (< 80 at.%). The alloys were produced by conventional casting (ingots) and melt-spinning (ribbons). Structural characterization was carried out for bulk ingots first, and then for the melt-spun ribbons. The ingots possessed a multiphase crystalline structure, as confirmed by X-ray diffraction and scanning electron microscopy observations. The amorphous structure of the melt-spun ribbons was determined by X-ray diffraction and transmission electron microscopy. SEM observations and EDX element maps of the cross-section of melt-spun ribbons indicated a homogeneous elemental composition. Neutron diffraction revealed the presence of nanocrystals in the amorphous matrix of the melt-spun ribbons. DSC data of the melt-spun ribbons showed exothermic events corresponding to the first crystallization at temperatures of 408 °C and 387 °C for Al_79_Ni_5_Fe_5_Y_11_ and Al_79_Ni_11_Fe_5_Y_5_, respectively.

## Introduction

The first aluminium-based metallic glasses were developed in the late 1980s by Inoue et al.^1^ and He et al.^2^ The discovery of Al-based alloys with disordered amorphous structures aroused great interest among scientists due to reports of their high strength and corrosion resistance^2,3^. However, their low glass-forming ability (GFA) connected with the requirement for high critical cooling rates to avoid crystallization has significantly limited the practical applications of Al-based metallic glasses^4^. Melt-spinning, due to its ability to obtain high cooling rates, is the most commonly used foundry technology enabling the production of amorphous aluminium alloys^4^. Shen and Perepezko^4^ explained that this is due to the low GFA of most Al-based alloy systems. In this method, a liquid metal is subjected to a very large cooling rate, which can reach 10^4^–10^9^ K/s. During the melt-spinning process, an ingot is placed in a ceramic crucible surrounded by an induction coil. Under the influence of alternating eddy currents, the temperature increases, which melts the charge material. The liquid metal is introduced onto a rapidly rotating copper wheel by a compressed gas^5,6^.

According to ref.^4^, the critical rate of cooling from the liquid state, which enables the glass transition of aluminium alloys, is 10^5^–10^6^ K/s. Therefore, these alloys are marginal glass formers or marginal metallic glasses. Despite their exceptional physical and chemical properties, the need to obtain them using high cooling rates limits the use of amorphous Al-based alloys to produce powders, wires, and ribbons. According to ref.^7^, the key issue is the stabilization of the supercooled liquid, which can be achieved by shifting the time-temperature-transformation curve responsible for the beginning of crystallization to lower cooling rates by increasing the time. According to ref.^7^, for this purpose, it is possible to minimize the amount of impurities in the alloy or to develop methods for designing chemical compositions with a high GFA, taking into account thermodynamic and kinetic aspects.

Amorphous structure in Al-based alloys was determined for the alloy systems Al–Y–Fe^8^, Al–Ni–Zr^9^, Al–Fe–Zr^9^, Al–Fe–Y^10,11^, Al–Y–Ni^3^, Al–Ni–Fe^12^, Al–Ni–Y^13,14^, Al–Ni–Fe–Gd^15^, and Al–Ni–Y–Co–Fe^16^. The most popular Al-based metallic glasses are three-component alloys with transition metals (TMs) and rare-earth elements (REs)^8,17^. According to the assumptions described in ref.^8,18^, the aluminium content should be in the range of 80–92 at.%, transition metals (TMs) 1–15 at.%, and rare earths (REs) 3–20%.

In this work, we present the results of structural studies of newly-developed Al_79_Ni_5_Fe_5_Y_11_ and Al_79_Ni_11_Fe_5_Y_5_ alloys. The aim of the article is to present new knowledge concerning Al-TMs-REs alloys with a reduced aluminium content produced by conventional casting and melt-spinning. The results presented in this paper will help facilitate the design of the chemical composition of Al-based metallic glasses. This paper contains the structural characteristics of slowly-cooled crystalline alloys in the form of ingots first and then rapidly-solidified amorphous ribbons cast by melt-spinning, as well as the results of differential scanning calorimetry.

## Materials and methods

Two newly-developed Al_79_Ni_5_Fe_5_Y_11_ and Al_79_Ni_11_Fe_5_Y_5_ alloys with a reduced aluminium content (< 80 at.%) were studied. Ingots of Al, Ni, Fe, and Y elements with a purity of 99.99% were melted in an induction furnace under a protective argon atmosphere in cylindrical corundum crucibles and then slowly cooled. The dimensions of the ingots were 50 mm high and 30 mm in diameter. The ingots produced were remelted and cast into ribbons by rapidly cooling from the liquid state by melt-spinning using a Bühler Melt Spinner SC station. The linear speed of the copper wheel with a diameter of 200 mm via the melt-spinning method was 30 m/s, which corresponds to a rotational speed of approximately 2865 rpm. The ribbon casting temperature was 1400 °C for Al_79_Ni_5_Fe_5_Y_11_ alloy and 1200 °C for Al_79_Ni_11_Fe_5_Y_5_ alloy. The melt-spun ribbons were approximately 50 μm thick and 10 mm wide.

X-ray diffraction (XRD) patterns were recorded using a Mini Flex 600 equipped with a copper tube Cu Kα (λ = 0.154 nm) as the X-ray radiation source and a D/TEX strip detector. The XRD patterns were collected in the Bragg–Brentano geometry. Samples in the form of ribbons were powdered for XRD measurements.

Neutron diffraction studies of alloys in the form of melt-spun alloys were performed on the MTEST neutron powder diffractometer at the Budapest Neutron Center. The powdered ribbons were measured in vanadium cans with a diameter of 6 mm. The Cu (111) monochromator selected neutrons with a wavelength of λ = 0.145 nm.

Observations of the microstructures were made using a Cs-corrected transmission electron microscope S/TEM Titan 80–300 from FEI Company. High-resolution transmission electron microscopy (HRTEM) imaging was also used. The diffraction patterns were obtained with both selected area diffraction (SAED) and Fourier transformations from HRTEM images. The melt-spun samples in the form of circles with a diameter of 3 mm were processed by a precise ion polishing system (Gatan 691).

The microstructures of alloy ingots were characterized by scanning electron microscopy (EVO MA10, Carl Zeiss) using the backscattered electron (BSE) mode. The cross-sections of melt-spun ribbons were observed using the secondary electron (SE) mode (Supra 35, Carl Zeiss). The maps of chemical elements were obtained by using energy-dispersive X-ray spectroscopy (EDX).

^57^Fe Mössbauer transmission spectra were recorded at room temperature with an MS96 Mössbauer spectrometer and a linearly-arranged ^57^Co:Rh source. Numerical analysis of the Mössbauer spectra was performed using the WMOSS program.

The crystallization mechanisms of the studied alloys in the form of ribbons were described using differential scanning calorimetry (DSC). Two temperature ranges were used: from 200 to 1000 °C by using a thermal analyzer SDT Q600 (Al_2_O_3_/Al_2_O_3_) and from 200 to 700 °C by a 910 model (DuPont Company (Pt/Pt)).

### Ethical approval

This article does not contain any studies with human participants or animals performed by any of the authors.

## Results and discussion

**Figure 1:**
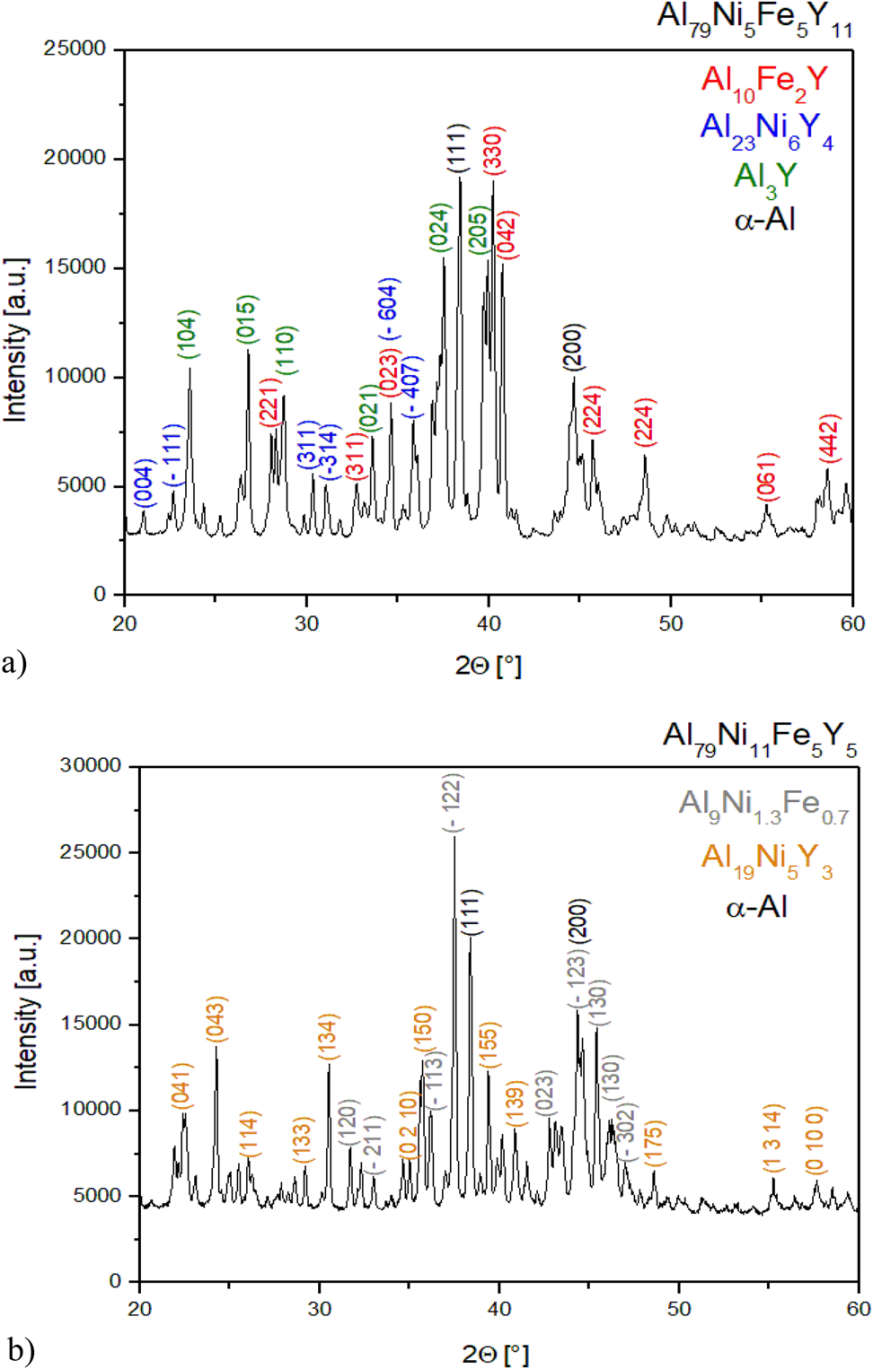
X-ray diffraction patterns of Al_79_Ni_5_Fe_5_Y_11_ (**a**) and Al_79_Ni_11_Fe_5_Y_5_ (**b**) in the form of ingots.

To identify the structure of Al_79_Ni_5_Fe_5_Y_11_ and Al_79_Ni_11_Fe_5_Y_5_ alloys in the form of slowly-cooled ingots, X-ray diffraction was carried out. Figure 1 shows the XRD patterns with Miller indices for the identified phases. The studied ingots possessed a multiphase crystalline structure. The following phases were identified for the Al_79_Ni_5_Fe_5_Y_11_ alloy: Al_10_Fe_2_Y, Al_3_Y, Al_23_Ni_6_Y_4_, and α-Al. In the structure of the Al_79_Ni_11_Fe_5_Y_5_ alloy, three phases were identified: Al_19_Ni_5_Y_3_, Al_9_Ni_1.3_Fe_0.7_, and α-Al.

The X-ray phase analysis of the Al_85_Ni_5_Fe_5_Y_5_ alloy, with a similar chemical composition to the studied alloys, is presented in ref.^19^. Similarly to the studied alloys, this alloy contained α-Al. In addition, Al_23_Ni_6_Y_4_ and Al_10_Fe_2_Y phases were identified in the Al_79_Ni_5_Fe_5_Y_11_ alloy. In ref.^20^, the authors proved that during annealing of the Al_86_Ni_8_Y_6_ alloy with an amorphous structure, the Al_23_Ni_6_Y_4_ phase crystallized first, followed by the α-Al phase due to the local depletion of nickel and yttrium in the metallic liquid. However, according to the research results described in ref.^21^, the phase crystallization sequence in the alloy with a similar chemical composition Al_87_Ni_9_Y_4_ is as follows: α-Al, Al_3_Ni, and Al_19_Ni_5_Y_3_. The Al_23_Ni_6_Y_4_ phase was identified in the Al_79_Ni_5_Fe_5_Y_11_ alloy, while the presence of the Al_19_Ni_5_Y_3_ phase was demonstrated for the Al_79_Ni_11_Fe_5_Y_5_ alloy. Similarly to ref.^20^, the Al_3_Ni phase was not identified, which should be present, according to the Al–Ni–Y phase equilibrium diagram. On the other hand, the Al_3_Y phase was identified only for the Al_79_Ni_5_Fe_5_Y_11_ alloy, which resulted from the higher atomic content of yttrium. Al–Fe phases were not found in the Al_79_Ni_11_Fe_5_Y_5_ alloy, probably due to the depletion of iron in the alloy after crystallization of the Al_9_Ni_1.3_Fe_0.7_ and Al_10_Fe_2_Y phases.

**Figure 2:**
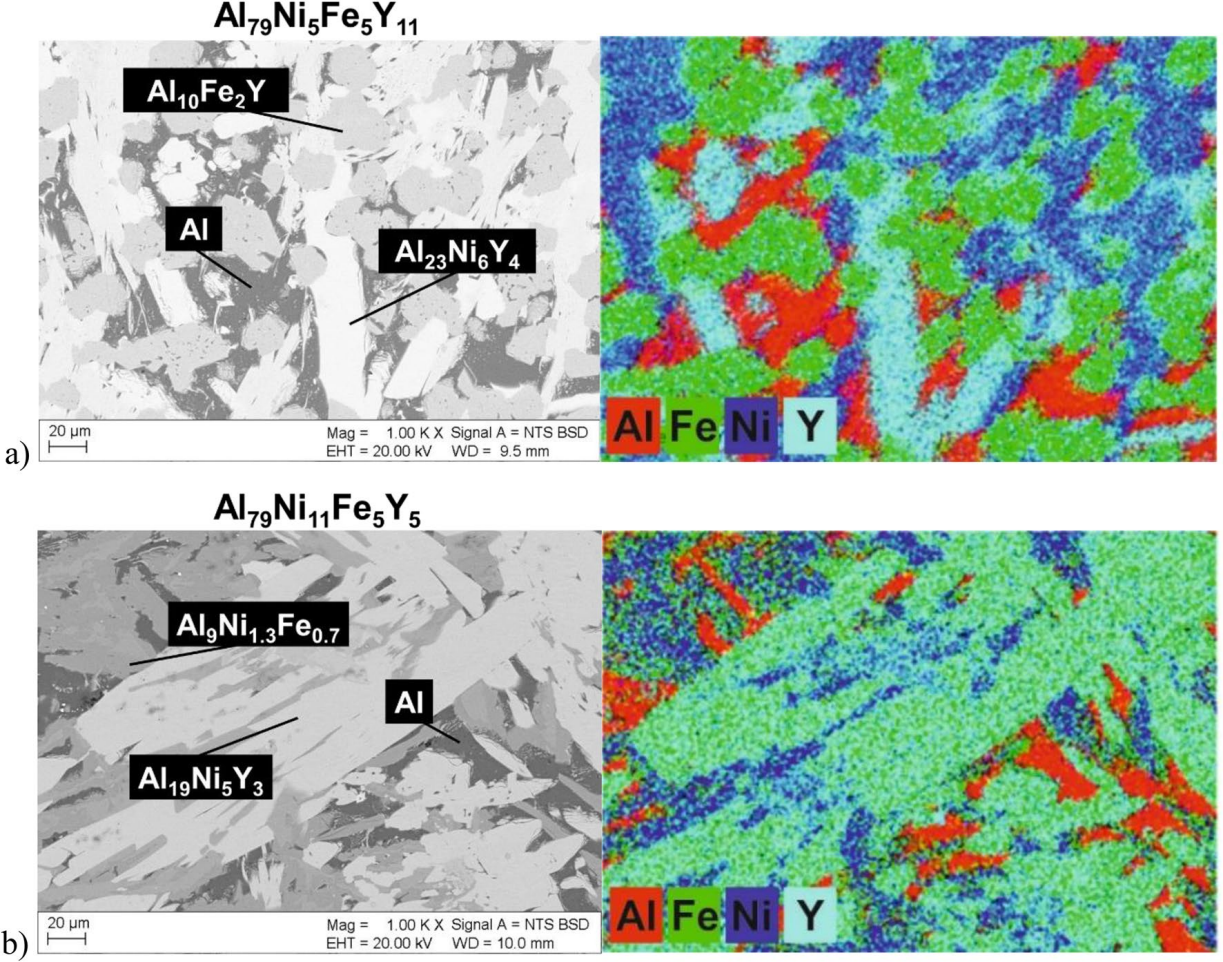
SEM images of the microstructures of Al_79_Ni_5_Fe_5_Y_11_ (**a**) and Al_79_Ni_11_Fe_5_Y_5_ (**b**) alloys in the form of ingots in BSE mode with element distribution maps.

The presence of a multiphase crystalline structure in the Al_79_Ni_5_Fe_5_Y_11_ and Al_79_Ni_11_Fe_5_Y_5_ ingots was confirmed by microstructure observations using SEM. The images of the microstructures in the backscattered electron (BSE) mode and the EDX element distribution maps are shown in Fig. 2. In the studied alloys, phases consisting of aluminium, nickel, and yttrium (Al_19_Ni_5_Y_3_ and Al_23_Ni_6_Y_4_) were present in the form of lamellar precipitates. The EDX maps confirmed the presence of the α-Al phase due to the presence of areas characteristic of aluminium (marked in red). This was confirmed by the presence of the α-Al phase, for which two high-intensity peaks were identified in the XRD patterns in studied ingots.

The microstructures of Al–Zr–Ni–Fe–Y alloys in the form of ingots are presented in ref.^22^, in which the Al_3_Y phase was present in the form of small platelets. However, the Al_3_Y phase in the SEM microstructures of Al-Y-Fe master alloys was observed in the form of longitudinal, regular precipitates in ref.^23^. The Al_10_Fe_2_Y phase in the Al_88_Y_8−x_Fe_4+x_ alloys (*x* = 0, 1, 2 at. %) formed a dendritic structure, while the α-Al phase was the matrix, which was characterized by the darkest shade in the SEM images in the studied alloys^23^. Xu et al.^24^ presented the microstructure of an Al–Ni–Y alloy produced using slow cooling and under pressure. Using both solidification methods, the Al_88_Ni_7_Y_5_ alloy consisted of α-Al, Al_3_(Ni,Y) and Al(Ni,Y) phases. The slowly cooled alloy was characterized by a structure consisting of thick plates of the Al_3_(Ni,Y) phase and thin needles of the Al(Ni,Y) phase. Cooling under a pressure of 6 GPa changed the coarse-grained Al_3_(Ni,Y) phase into branched dendrites^24^. The presence of thick, lamellar precipitates of the Al–Ni–Y phases was also observed in the studied alloys. Similar microstructures were also presented in ref.^25^ for the multiphase Al_85_Ni_7_Fe_4_La_4_ alloy in the form of an ingot. The α-Al phase, similar to the studied alloys, was identified as the darkest precipitates. In ref.^25^, the authors indicated the phase marked Al_9_Ni_1−x_Fe_x_ as oblong, oriented along one direction of the plate. In the case of the studied alloys, the largest number of reflections for the Al_9_Ni_1.3_Fe_0.7_ phase was identified in the XRD patterns of the Al_79_Ni_11_Fe_5_Y_5_ alloy. Based on the EDX maps, the Al_9_Ni_1.3_Fe_0.7_ phase was marked in the SEM image as medium-gray precipitates. In contrast to the microstructure described in ref.^25^, an orientation along one direction was not observed for the Al_9_Ni_1.3_Fe_0.7_ phase.

**Figure 3:**
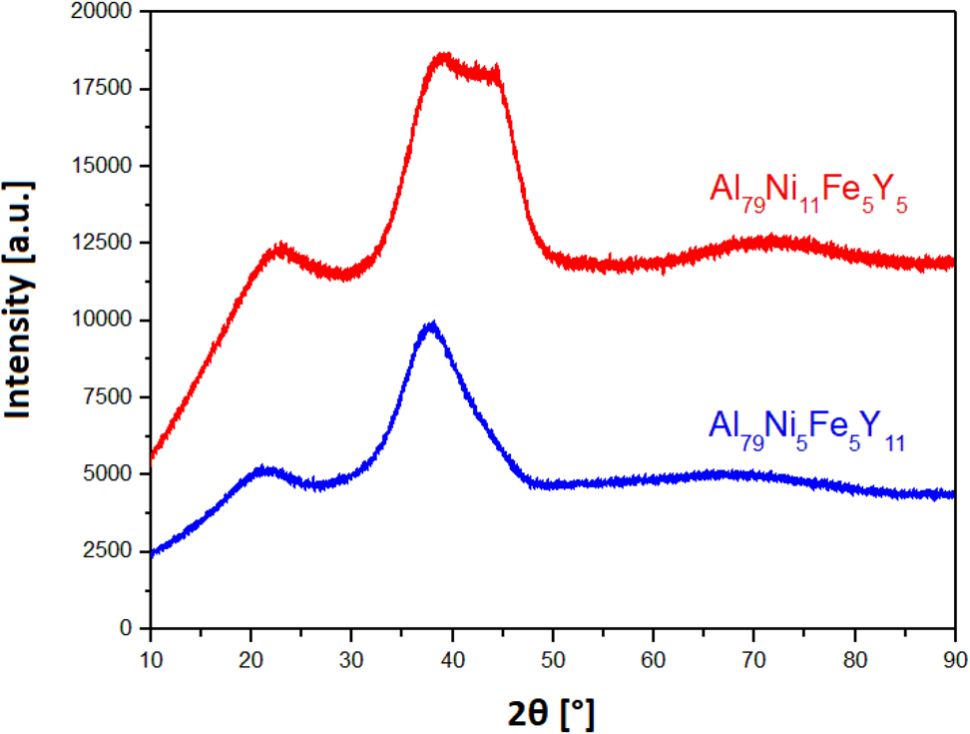
X-ray diffraction patterns of Al_79_Ni_11_Fe_5_Y_5_ and Al_79_Ni_5_Fe_5_Y_11_ in the form of ribbons.

Figure 3 presents the XRD patterns of melt-spun Al_79_Ni_5_Fe_5_Y_11_ and Al_79_Ni_11_Fe_5_Y_5_ alloys, which indicates an amorphous structure because of the characteristic amorphous “halo” and the lack of crystalline reflections. However, the XRD pattern of Al_79_Ni_11_Fe_5_Y_5_ was characterized by a broad peak that indicated a double-amorphous state.

According to ref.^20^, the Al_23_Ni_6_Y_4_ phase limits the vitrification of Al–Ni–Y alloys because it was identified as the primary phase during the crystallization of the amorphous Al_86_Ni_8_Y_6_ alloy. However, in the case of the Al_79_Ni_5_Fe_5_Y_11_ alloy in the form of an ingot, the Al_23_Ni_6_Y_4_ phase was identified. An amorphous structure was obtained for the ribbons with this chemical composition. According to previous literature^8,17^, Al-TM-RE alloys with a disordered atomic structure should contain 80–92 at.% aluminium. Despite the reduced content of the main alloying element, an amorphous structure was obtained for the Al_79_Ni_11_Fe_5_Y_5_ and Al_79_Ni_5_Fe_5_Y_11_ alloys.

**Figure 4:**
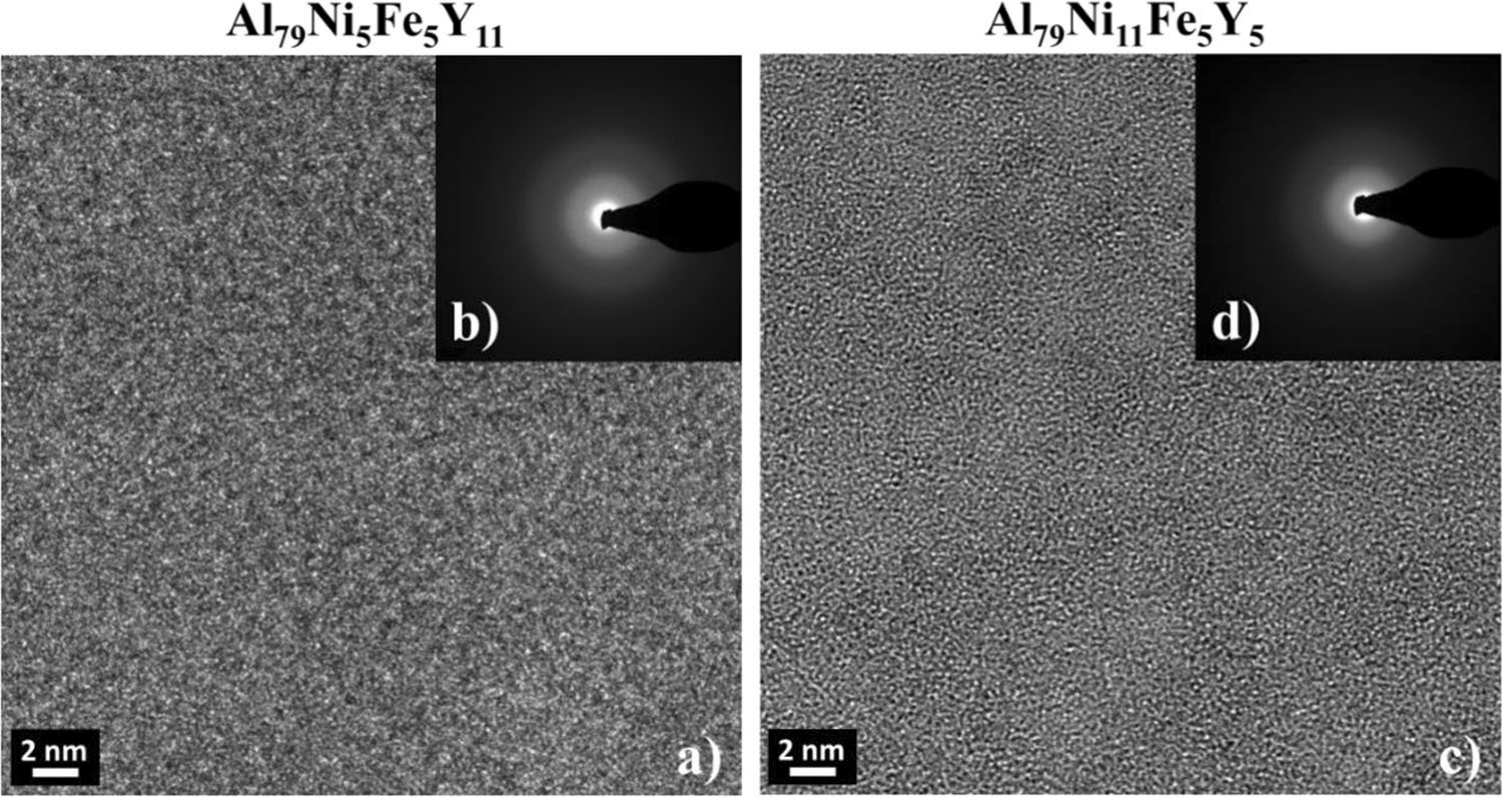
HRTEM images (**a**, **c**) and SAED patterns (**b**, **d**) of the Al_79_Ni_5_Fe_5_Y_11_ and Al_79_Ni_11_Fe_5_Y_5_ alloys in the form of melt-spun ribbons.

To confirm the amorphous structure of Al_79_Ni_5_Fe_5_Y_11_ and Al_79_Ni_11_Fe_5_Y_5_ alloys in the form of melt-spun ribbons, TEM observations were carried out. Figure 4 shows the HRTEM images and selected area electron diffractions (SAED) pattern. The microscopic observations showed that the studied alloys were characterized by a homogeneous structure devoid of crystallites. Structures in the high-resolution mode were characterized by atomic disorder, referred to in the literature^26^ as the “salt and pepper” effect. In addition, the presence of an amorphous structure for the Al_79_Ni_5_Fe_5_Y_11_ and Al_79_Ni_11_Fe_5_Y_5_ alloys was confirmed by the SAED results due to the broadened ring patterns.

**Figure 5:**
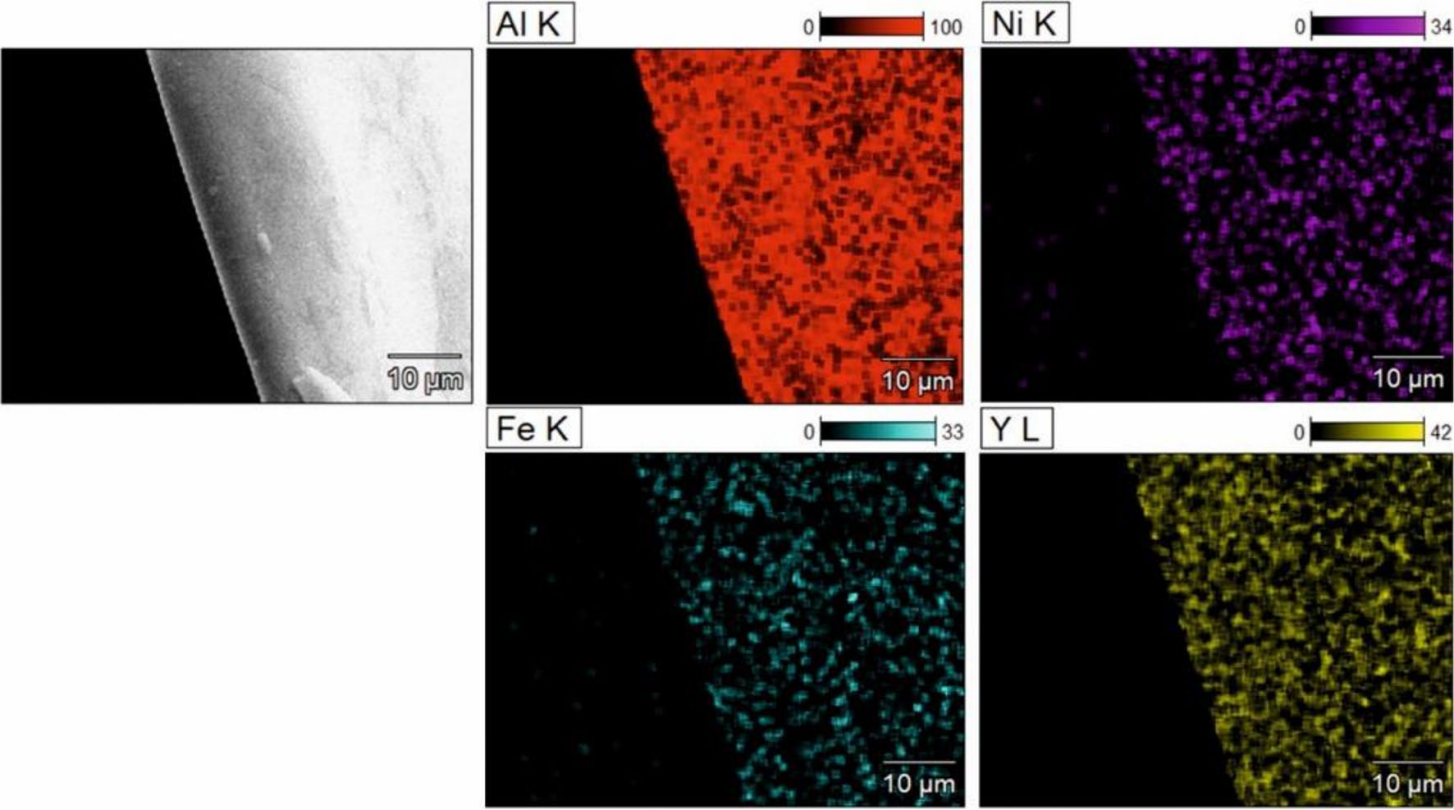
Microstructure of Al_79_Ni_5_Fe_5_Y_11_ alloy in ribbon form with EDX element distribution maps.

**Figure 6:**
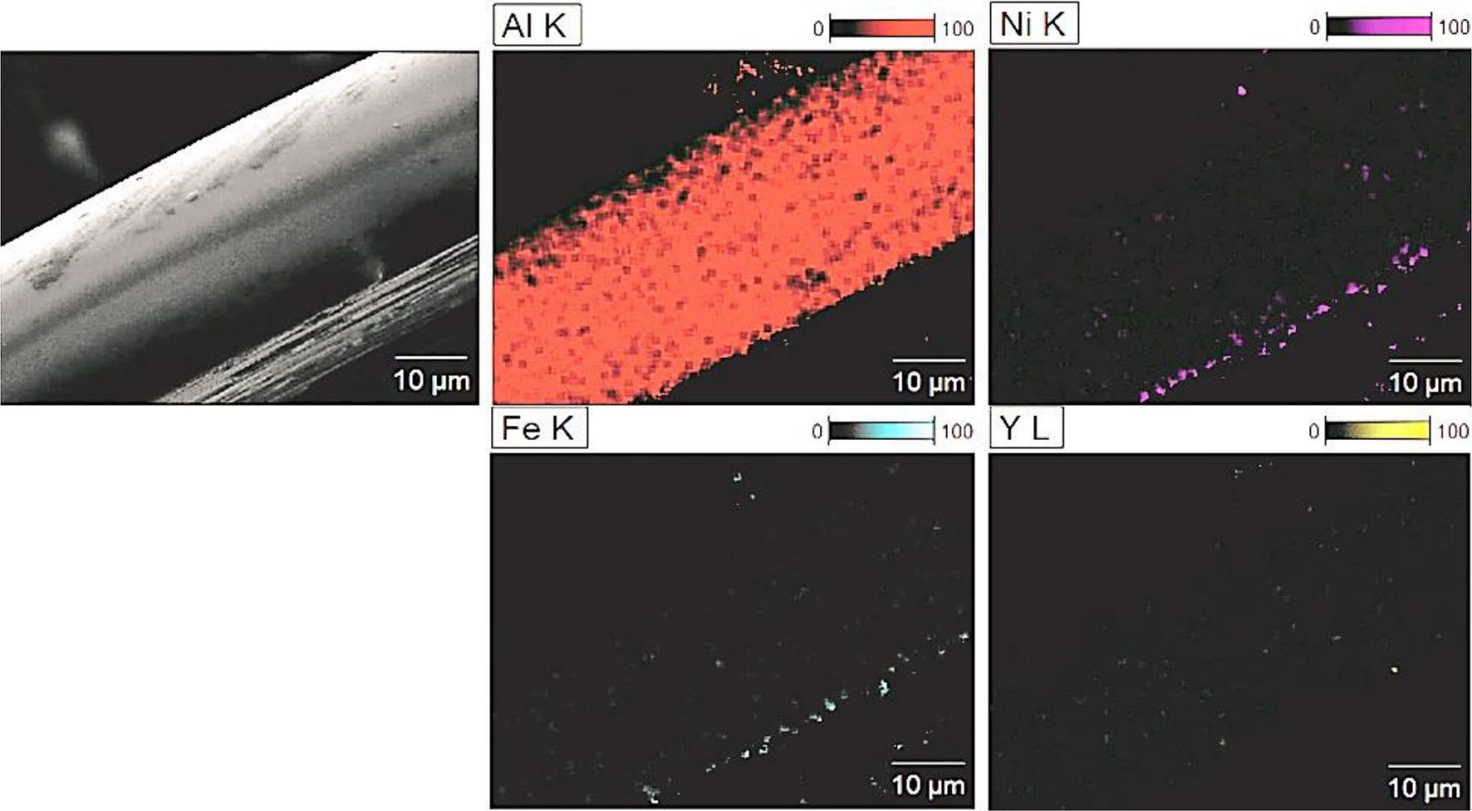
Microstructure of Al_79_Ni_11_Fe_5_Y_5_ alloy in ribbon form with EDX element distribution maps.

The elemental distributions were collected for Al_79_Ni_5_Fe_5_Y_11_ (Fig. 5) and Al_79_Ni_11_Fe_5_Y_5_ (Fig. 6) alloys in ribbon form. The external morphology of the samples was also obtained in SE mode. The alloys were assigned as homogeneous single-phase structures with no segregation. It can be seen that the maps presented areas with different concentrations of Al, Ni, Fe, and Y elements according to the nominal chemical compositions of the samples. The homogeneous concentration of the elements also confirmed the amorphous structure of the tested ribbons. Previous works^27,28^ have reported that an amorphous structure was obtained on the surface as a result of contact with the copper wheel during the melt-spinning process. The influence of material surface contact during cooling on the structure was described, i.e., for Ti–Ni–Cu alloys produced by the melt-spinning method. An amorphous zone (called the contact surface) and the crystalline zone (called the free surface) were visible on the cross-sectional SEM images of the studied ribbons^27^.

**Figure 7:**
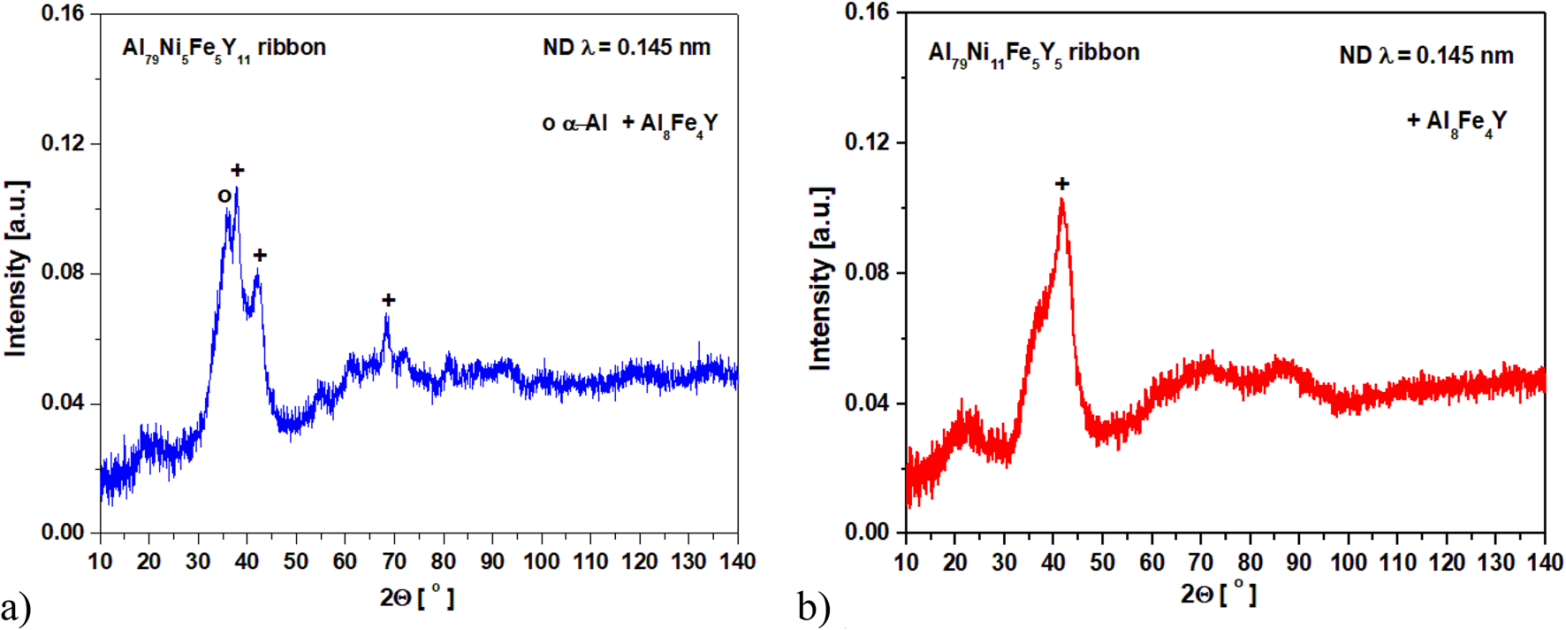
Neutron diffraction patterns of Al_79_Ni_5_Fe_5_Y_11_ (**a**) and Al_79_Ni_11_Fe_5_Y_5_ (**b**) alloys in the form of ribbons.

Figure 7 shows the neutron diffraction patterns of the alloys in the form of melt-spun ribbons. Broad diffraction peaks indicating the reflections of crystallites were observed for both alloys. The reflections for the α-Al and Al_8_Fe_4_Y phases were identified for the Al_79_Ni_5_Fe_5_Y_11_ alloy, while reflections for the Al_8_Fe_4_Y phase were observed for the Al_79_Ni_11_Fe_5_Y_5_ alloy. The penetration depth of neutrons in matter is much deeper compared with X-rays and electrons; therefore, according to ref.^29^, neutron radiation is useful for studying bulk materials. According to literature data^30,31^, the amorphous structure of metallic glasses should make them resistant to irradiation. Yang et al.^30^ studied the structural responses of ZrCu metallic glasses under neutron irradiation and did not observe the formation of any crystalline phase, even though they confirmed its presence by synchrotron-based high-energy X-ray diffraction. However, in the same article^30^, the mechanisms of neutron irradiation that damaged the microstructure of amorphous alloys remained elusive. In this study, the melt-spun ribbons likely had a heterogeneous structure.

**Figure 8:**
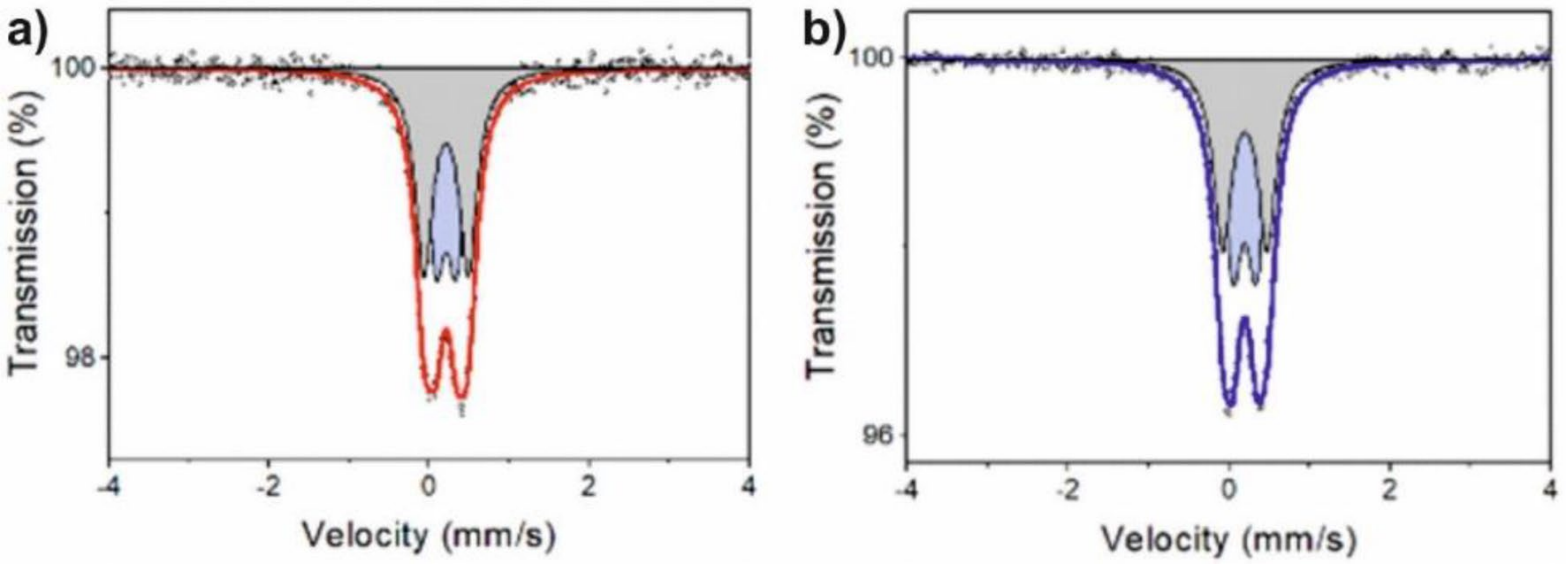
Mössbauer spectra of Al_79_Ni_5_Fe_5_Y_11_ (**a**) and Al_79_Ni_11_Fe_5_Y_5_ alloys (**b**).

Mössbauer spectra with their adjustments obtained for the Al_79_Ni_5_Fe_5_Y_11_ and Al_79_Ni_11_Fe_5_Y_5_ alloys in the form of ribbons are shown in Fig. 8. These spectra were fitted with non-magnetic components (quadrupole doublets)^32^. The determined hyperfine parameters of these components are summarized in Table 1. The spectrum of the Al_79_Ni_11_Fe_5_Y_5_ alloy contained two doublets, indicating the presence of two different local environments of iron atoms. The isomeric shifts (*I*s) of both these doublets were similar and in the range of 0.19–0.21 mm/s, but these components differed significantly in their quadrupole splitting (*Q*s). The *Qs* range was 0.25–0.29 mm/s for the first doublet, and 0.54–0.56 mm/s for the second. Taking into account the XRD results for these alloys, we can associate these doublets with the presence of iron in the aluminium-rich amorphous structure^33^. The diffraction pattern of the Al_79_Ni_5_Fe_5_Y_11_ alloy in the form of ribbons indicated the presence of an amorphous structure, while that of the Al_79_Ni_11_Fe_5_Y_5_ alloy was characterized by a double “halo,” indicating the presence of two types of atomic disorder. The significantly different quadrupole splitting values of these two doublets resulted from different local geometries of the distributions of nickel and yttrium atoms around iron atoms^23^. The component with higher values of quadrupole splitting will be associated with iron atoms having mainly aluminium and yttrium in their local environments, and the doublet with lower *Q*s values will be associated with iron atoms surrounded mainly by aluminium and nickel atoms. Yttrium atoms with a larger atomic radius than nickel atoms caused greater distortion of the local iron environment, hence higher values of quadrupole splitting.

**Table 1:** The Mössbauer hyperfine parameters of the investigated samples.

Alloy	*I*s (mm/s)	*Q*s (mm/s)	FWHM (mm/s)	*A* (%)	Phase
Al_79_Ni_5_Fe_5_Y_11_	0.21	0.25	0.27	47	Rich in Al, Fe, Y
	0.21	0.55		53	Rich in Al, Fe, Ni
	0.21*	0.42*	0.26	–	–
Al_79_Ni_11_Fe_5_Y_5_	0.19	0.28	0.28	51	Rich in Al, Fe, Y
	0.20	0.56		49	Rich in Al, Fe, Ni
	0.20*	0.41*	0.26*	–	–

*Is* - isomer shift, *Qs* - quadrupole splitting, *FWHM* - full width at half maximum, *A* - relative area from the spectra, * - parameters related to p(*Q*s) of Al_79_Ni_5_Fe_5_Y_11_ and Al_79_Ni_11_Fe_5_Y_5_ alloys in the form of ribbons.

**Figure 9:**
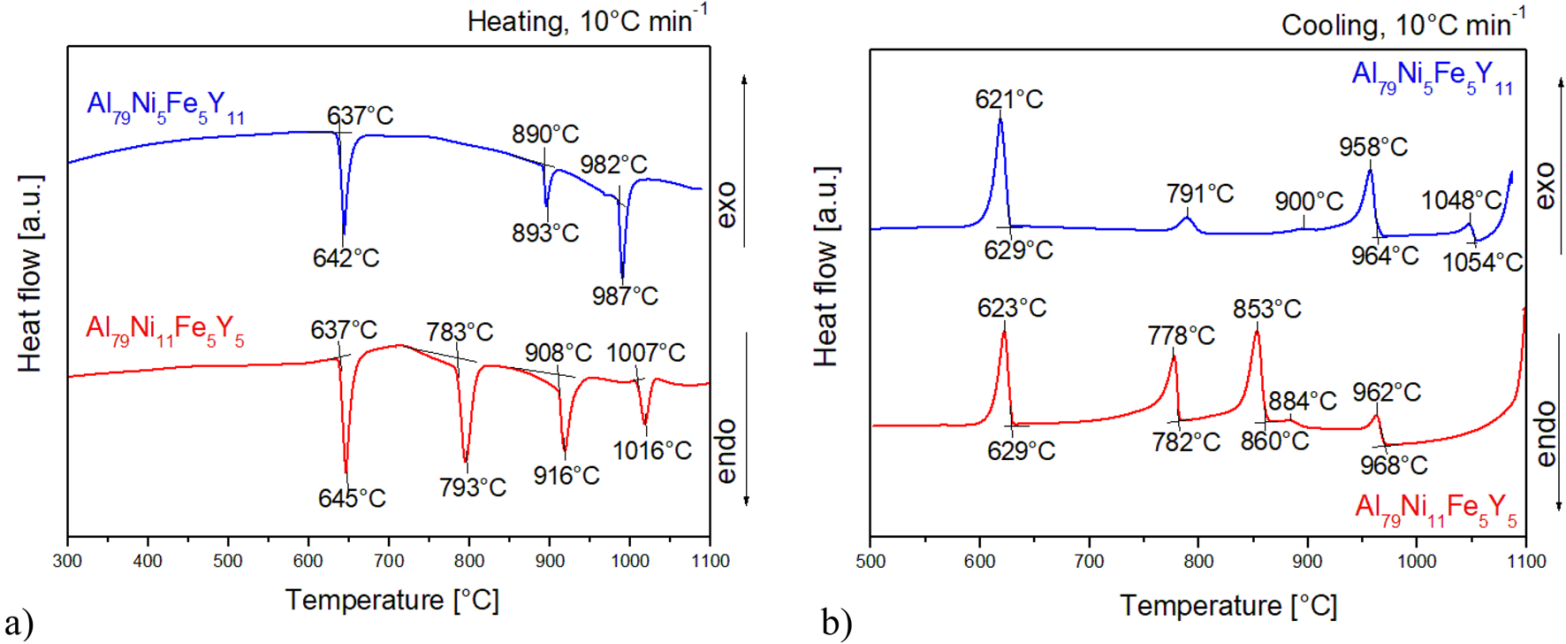
DSC heating (**a**) and cooling (**b**) curves of Al_79_Ni_5_Fe_5_Y_11_ and Al_79_Ni_11_Fe_5_Y_5_ alloys in the form of ingots.

To describe the thermal events under heating and cooling, the crystalline alloys (in the form of ingots) and amorphous ribbons were analyzed using DSC. As seen in Fig. 9, the DSC curve of the Al_79_Ni_5_Fe_5_Y_11_ ingot showed three endothermic peaks at 637 °C, 890 °C and 982 °C during heating from room temperature to 1100 °C, indicating the occurrence of phase transformations. On the other hand, during cooling from 1100 °C to room temperature, five exothermic peaks were observed at 1048 °C, 958 °C, 900 °C, 791 °C and 621 °C. The DSC curve of the Al_79_Ni_11_Fe_5_Y_5_ ingot showed four endothermic peaks at 637 °C, 783 °C, 908 °C and 1007 °C, while during cooling, five exothermic peaks were observed at 962 °C, 884 °C, 853 °C, 778 °C and 623 °C. According to literature data^19,22,34^, the thermal event above 600 °C probably corresponded to the melting of the α-Al phase during heating and its crystallization during cooling.

**Figure 10:**
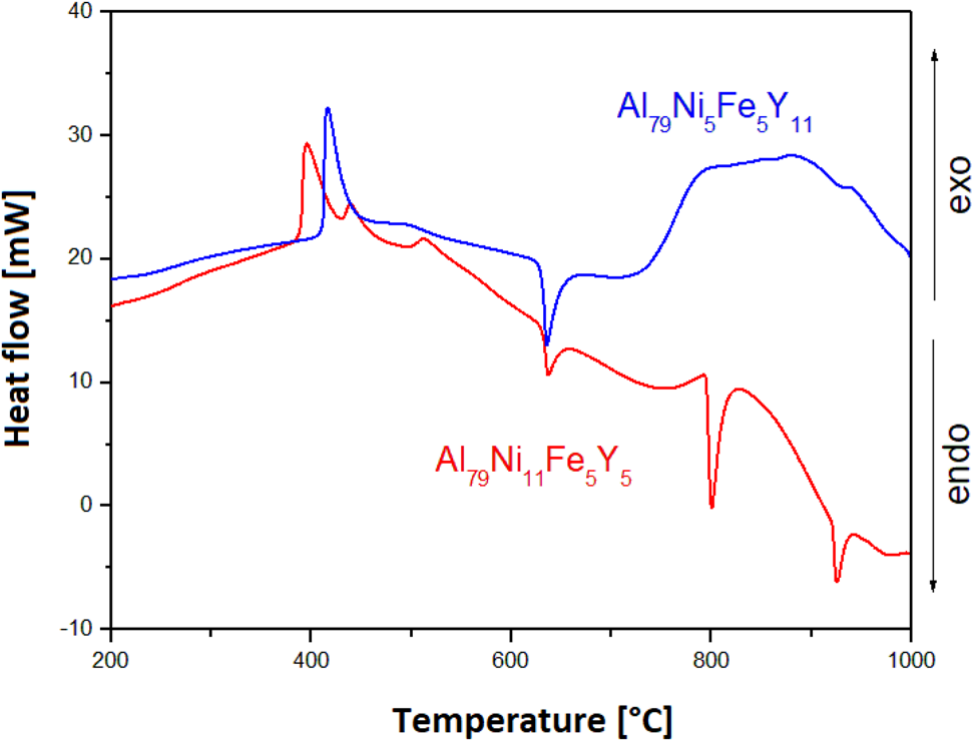
DSC heating curves of Al_79_Ni_5_Fe_5_Y_11_ and Al_79_Ni_11_Fe_5_Y_5_ alloys in the form of melt-spun ribbons.

**Figure 11:**
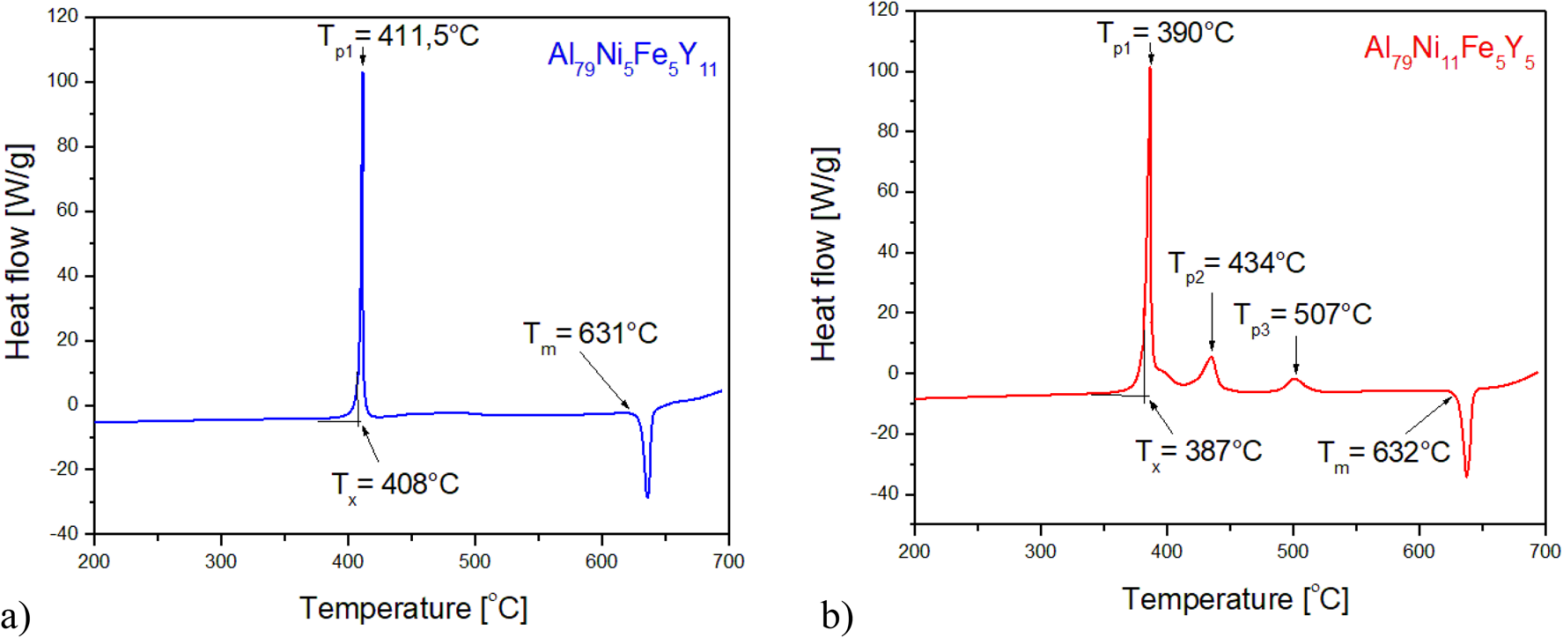
DSC heating curves of Al_79_Ni_5_Fe_5_Y_11_ (**a**) and Al_79_Ni_11_Fe_5_Y_5_ (**b**) alloys in the form of melt-spun ribbons.

Figure 10 shows the DSC curves recorded in the temperature range of 200–1000 °C at the rate of 10 °C/min during heating for Al_79_Ni_5_Fe_5_Y_11_ and Al_79_Ni_11_Fe_5_Y_5_ alloys in the form of ribbons. Figure 11 also shows the DSC curves for the same ribbons over a smaller temperature range (200–700 °C) and with a higher heating rate (20 °C/min). In the DSC curves (Fig. 10), three exothermic events appeared during heating at 390 °C, 433 °C, and 499 °C for Al_79_Ni_11_Fe_5_Y_5_ ribbon. Moreover, three endothermic events at 630 °C, 796 °C and 922 °C were also recorded. The DSC curve of the Al_79_Ni_5_Fe_5_Y_11_ ribbon showed one clear exothermic peak recorded at 412 °C as well as endothermic event at 630 °C. An endothermic reaction with a low enthalpy was also recorded at 900 °C. The recorded baseline for the ribbon with a higher yttrium content was characterized by an unstable course, probably related to the movement of metallic liquid in the measuring crucible. Based on the DSC curves shown in Fig. 11, *T*_x_ (onset crystallization temperature), *T*_p_ (crystallization peak temperature), and *T*_m_ (melting temperature) were determined. The Al_79_Ni_11_Fe_5_Y_5_ alloy was characterized by a lower temperature at the beginning of crystallization of the amorphous phase (*T*_x_ = 390 °C) compared with Al_79_Ni_5_Fe_5_Y_11_ alloy (*T*_x_ = 408 °C). Similarly to Fig. 10, two additional exothermic events were observed for the Al_79_Ni_11_Fe_5_Y_5_ alloy with crystallization temperatures of 434 °C and 507 °C. The Al_79_Ni_11_Fe_5_Y_5_ (*T*_m_ = 632 °C) and Al_79_Ni_5_Fe_5_Y_11_ (*T*_m_ = 631 °C) alloys were characterized by similar melting onset temperatures determined from the recorded endothermic event.

A similar course of the DSC curve as the Al_79_Ni_11_Fe_5_Y_5_ ribbons was presented in ref.^35^ for the Al_84.5_Ni_5.5_Y_10_ alloy with an amorphous structure in the form of a high-pressure cast rod. In ref.^35^, three distinct, consecutive exothermic events and one endothermic event were recorded. Fu et al.^35^ stated that the exothermic peaks corresponded to the crystallization of the amorphous structure, while the endothermic event was related to the glass transition. However, on the basis of ref.^4,36^ most of Al-based metallic glasses do not show a clear *T*_g_ glass-transition event because the onset of the *T*_x_ primary crystallization peak almost coincides with the glass transition. Moreover, according to the authors of ref.^35^, the liquid phase was present in practically the entire volume of the alloy after the first endothermic peak. On the basis of the DSC curve in ref.^35^, the glass transition temperature (*T*_g_), onset crystallization temperature (*T*_x1_), melting point (*T*_m_), and liquidus temperature (*T*_l_) were determined, respectively, as 207 °C, 244 °C, 617 °C, and 959 °C. Exothermic effects recorded for the Al_79_Ni_11_Fe_5_Y_5_ alloy occurred at higher temperatures due to differences in the chemical composition of the amorphous phase. In addition, two additional endothermic effects were observed in the DSC curve for the Al_79_Ni_11_Fe_5_Y_5_ alloy. The crystallization mechanisms of Al_85_Ni_10_Y_5_ and Al_85_Ni_5_Fe_5_Y_5_ alloys are described in ref.^34^ based on the XRD patterns obtained in situ at variable temperatures and the results of differential thermal analysis (DTA). Similarly to the Al_79_Ni_5_Fe_5_Y_11_ and Al_79_Ni_11_Fe_5_Y_5_ alloys, exothermic effects related to the crystallization of the amorphous phase were recorded on the DTA curves. On the basis of the diffractograms, it was estimated that in the Al_85_Ni_10_Y_5_ alloy, after the formation of the α-Al phase, the Al_19_Ni_5_Y_3_ phase crystallized at 340 °C. The temperature of 400 °C was associated with the crystallization of the Al_15_Fe_9_Y_2_ phase in both the Al_85_Ni_10_Y_5_ and Al_85_Ni_5_Fe_5_Y_5_ alloys. According to ref.^34^, the last stage was the crystallization of the AlNiY and Fe_0.7_Ni_1.3_Al_9_ phases.

## Conclusions

The ingots possessed a multiphase crystalline structures. Melt-spinning method was used to obtain supercooled alloys in the form of ribbons. The amorphous structure of the ribbons was confirmed by XRD, SEM, and TEM, however the results of neutron diffraction studies indicate that the melt-spun alloys exhibited amorphous matrix structure with the presence of crystalline phases. The diffraction pattern of the Al_79_Ni_5_Fe_5_Y_11_ ribbon indicated the presence of an amorphous structure, while the Al_79_Ni_11_Fe_5_Y_5_ alloy was characterized by a double-broadened peak that indicated the presence of two types of atomic disorder. Furthermore, exothermic events in the DSC curves indicated the occurrence of crystallization from the amorphous phase at 408 °C for the Al_79_Ni_5_Fe_5_Y_11_ alloy and at 387 °C for the Al_79_Ni_11_Fe_5_Y_5_ alloy.

## Data Availability

The data and material generated during and/or analyzed during the current study are available from the corresponding author upon reasonable request.
